# Supplementary material for: C-X-C Motif Chemokine Ligand 9 and Its CXCR3 Receptor Are the Salt and Pepper for T Cells Trafficking in a Mouse Model of Gaucher Disease
Source: Int J Mol Sci. 2021 Nov 24;22(23):12712. doi: 10.3390/ijms222312712 (PMC8657559; doi:10.3390/ijms222312712)
Supplement: Supplementary file 1 [file ijms-22-12712-s001.zip › ijms-1344586-supplementary.pdf]

International Journal of
Molecular Sciences

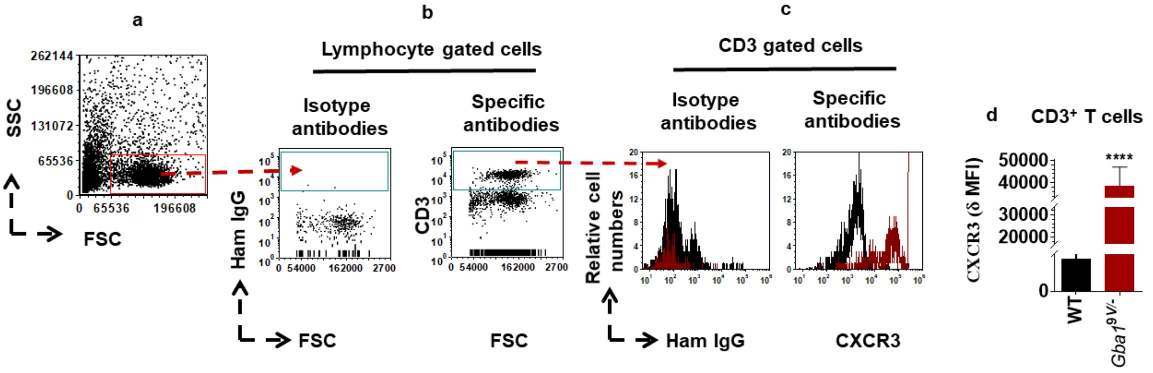

Supplementary Figure S1 a - d. CXCR3 surface expression in circulatory T cells from *Gba1*$^{9V/-}$ mice. CXCR3 expression in lymphocyte gated CD3^{+} T cells (a-d) from blood of strain-matched *Gba1*$^{9V/-}$ and WT mice (n=5/group). δ MFI = CXCR3 MFI - isotype MFI. In the histograms of isotypes (c), specific antibodies (d), and the bar diagram (d), the black lines/columns correspond to WT and the maroon lines/columns to *Gba1*$^{9V/-}$ mice. Values in d are the means ± s. d. and asterisks show significant differences between WT and *Gba1*$^{9V/-}$ mice (****p<0.0001). Three independent experiments were conducted and groups were compared using student's *t*-tests.

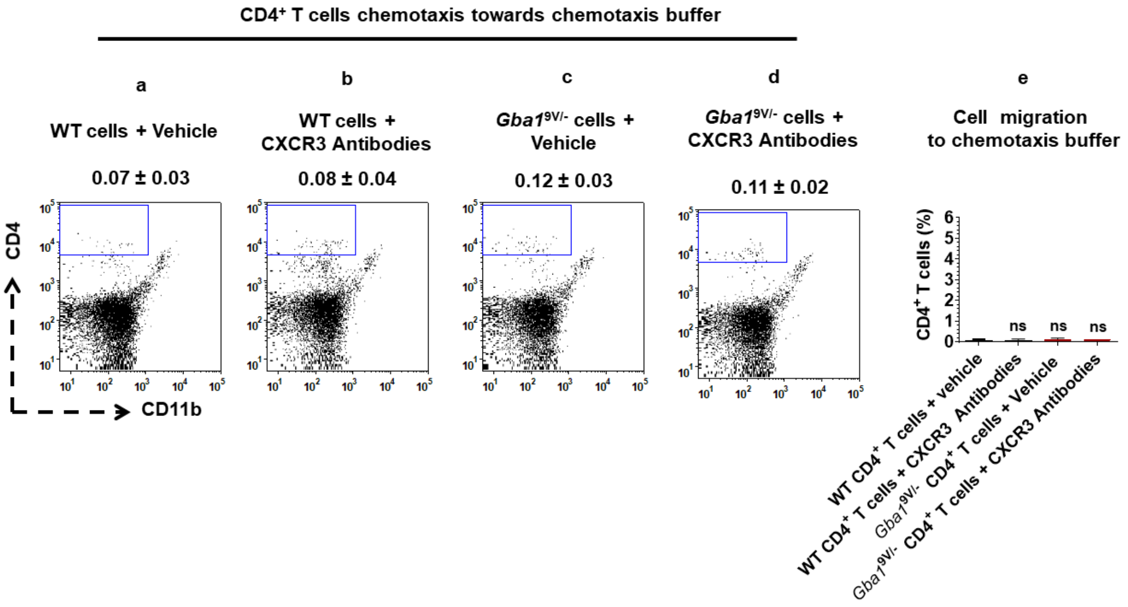

Supplementary. Figure S2 a - e. CXCR3 blocking and CD4⁺ T cells chemotaxis towards 2% Gey's Balanced Salt Solution (GBSS; chemotaxis buffer). CD4^{+}T cells purified from spleens of WT and *Gba1*$^{9V/-}$ mice (n=5/group) were allowed to migrate towards 2% Gey's Balanced Salt Solution (GBSS; chemotaxis buffer).in the presence and absence of antibodies to mouse CXCR3 at 37°C and 5% CO2 for 45 minutes as described in the method. Cells that had migrated through the filter and had attached to the lower side of the filter were collected and analyzed by FACS. Percentage of CD4^{+}CD11b^{-} T cells are shown from the (a) vehicle (PBS) treated WT cells and their migration to chemotaxis buffer, (b) CXCR3 antibody treated WT cells and their migration to chemotaxis buffer, (c) PBS treated *Gba1*$^{9V/-}$ cells and their migration to chemotaxis buffer, and (d) CXCR3 antibody treated *Gba1*$^{9V/-}$ cells and their migration to chemotaxis buffer. (e) WT (black columns), *Gba1*$^{9V/-}$ (Maroon columns), and the values shown in the bar diagram are the mean ± s. d. and group comparison were performed with ANOVA. Three independent experiments were conducted (ns, not significant).

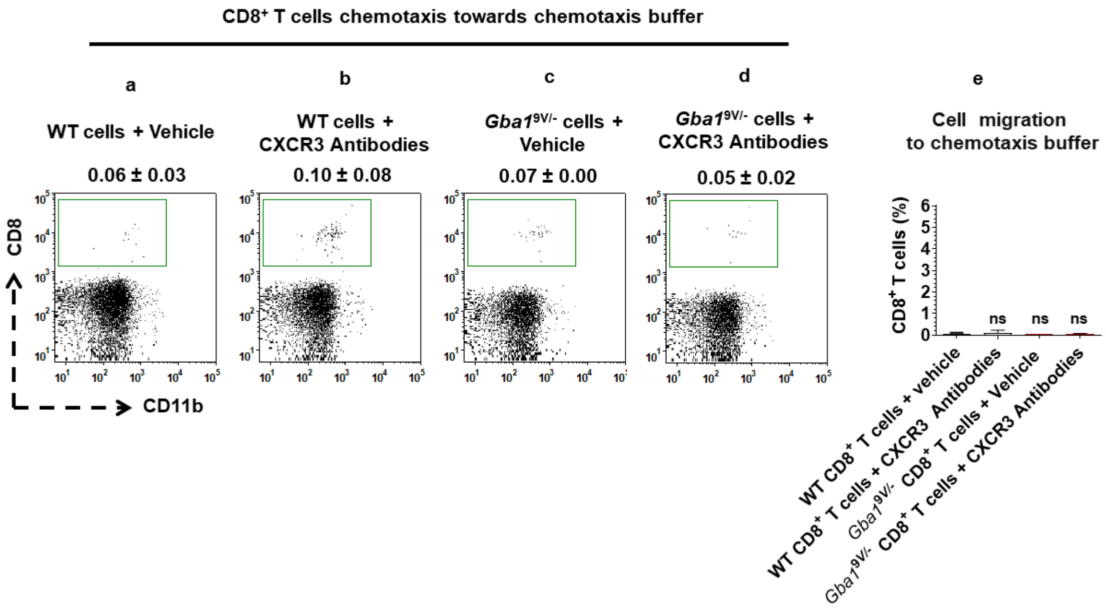

Supplementary. Figure S3 a - e. CXCR3 blocking and $CD8^+$ T cells chemotaxis towards 2% Gey's Balanced Salt Solution (GBSS; chemotaxis buffer). $CD8^+$T cells purified spleens of WT and *Gba1* $^{9V/-}$ mice (n=5/group) were allowed to migrate towards 2% Gey's Balanced Salt Solution (GBSS; chemotaxis buffer).in the presence and absence of antibodies to mouse CXCR3 at 37 ^{o}C and 5% CO2 for 45 minutes as described in the method. Cells that had migrated through the filter and had attached to the lower side of the filter were collected and analyzed by FACS. Percentage of $CD8^+CD11b^-$ T cells are shown from the (a) vehicle (PBS) treated WT cells and their migration to chemotaxis buffer, i.e., 2% Gey's Balanced Salt Solution **(**GBSS), (b) CXCR3 antibody treated WT cells and their migration to chemotaxis buffer, (c) PBS treated *Gba1* $^{9V/-}$ cells and their migration to chemotaxis buffer, and (d) CXCR3 antibody treated *Gba1* $^{9V/-}$ cells and their migration to chemotaxis buffer. (e) WT (black columns), *Gba1* $^{9V/-}$ (Maroon columns), and values shown in the bar diagram are the mean ± s. d. and group comparison were performed with ANOVA. Three independent experiments were conducted (ns, not significant).

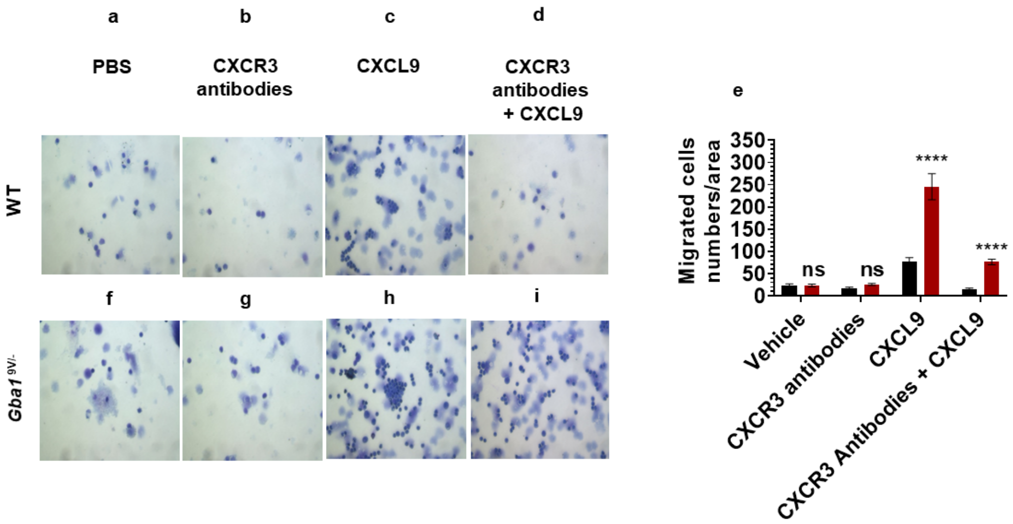

Supplementary. Figure S4 a - i. In vivo blocking of CXCR3 and microscopic evaluation of CXCL9- mediated peritoneal cell infiltrates in *Gba1* $^{9V/-}$ mice. WT and *Gba1* $^{9V/-}$ mice were injected with intraperitoneal administration of CXCL9 (c and h), antibodies to mouse CXCR3 (b and g), and its vehicle (a and f). In additional experiments, these mice were injected with intravenous injection of antibodies to mouse CXCR3 prior to intraperitoneal injection of CXCL9 (d and i). The peritoneal cells were stained with Diff-Quick staining and cells were counted under the light microscope. The corresponding bar diagrams shown on right represent the peritoneal cells migration to CXCL9 in the presence or absence of vehicle and antibodies to CXCR3 in WT and the *Gba1* $^{9V/-}$ mice (e). WT (black columns), *Gba1* $^{9V/-}$ (Maroon

columns), and the values shown are the mean ± s. d. and group comparison were performed with ANOVA (ns, not significant; ****, $p<0.0001$).
